# Supplementary material for: Pumilio proteins license viral noncoding RNA engagement with target mRNAs
Source: bioRxiv. 2026 Oct 7:2026.10.06.757068. Preprint. [Version 1] doi: 10.64898/2026.10.06.757068 (PMC13692250; doi:10.64898/2026.10.06.757068)
Supplement: Supplementary file 1 [file NIHPP2026.10.06.757068v1-supplement-1.pdf]

bioRxiv preprint doi: https://doi.org/10.64898/2026.10.06.757068; this version posted October 7, 2026. The copyright holder for this preprint (which was not certified by peer review) is the author/funder, who has granted bioRxiv a license to display the preprint in perpetuity. It is made available under a CC-BY 4.0 International license.

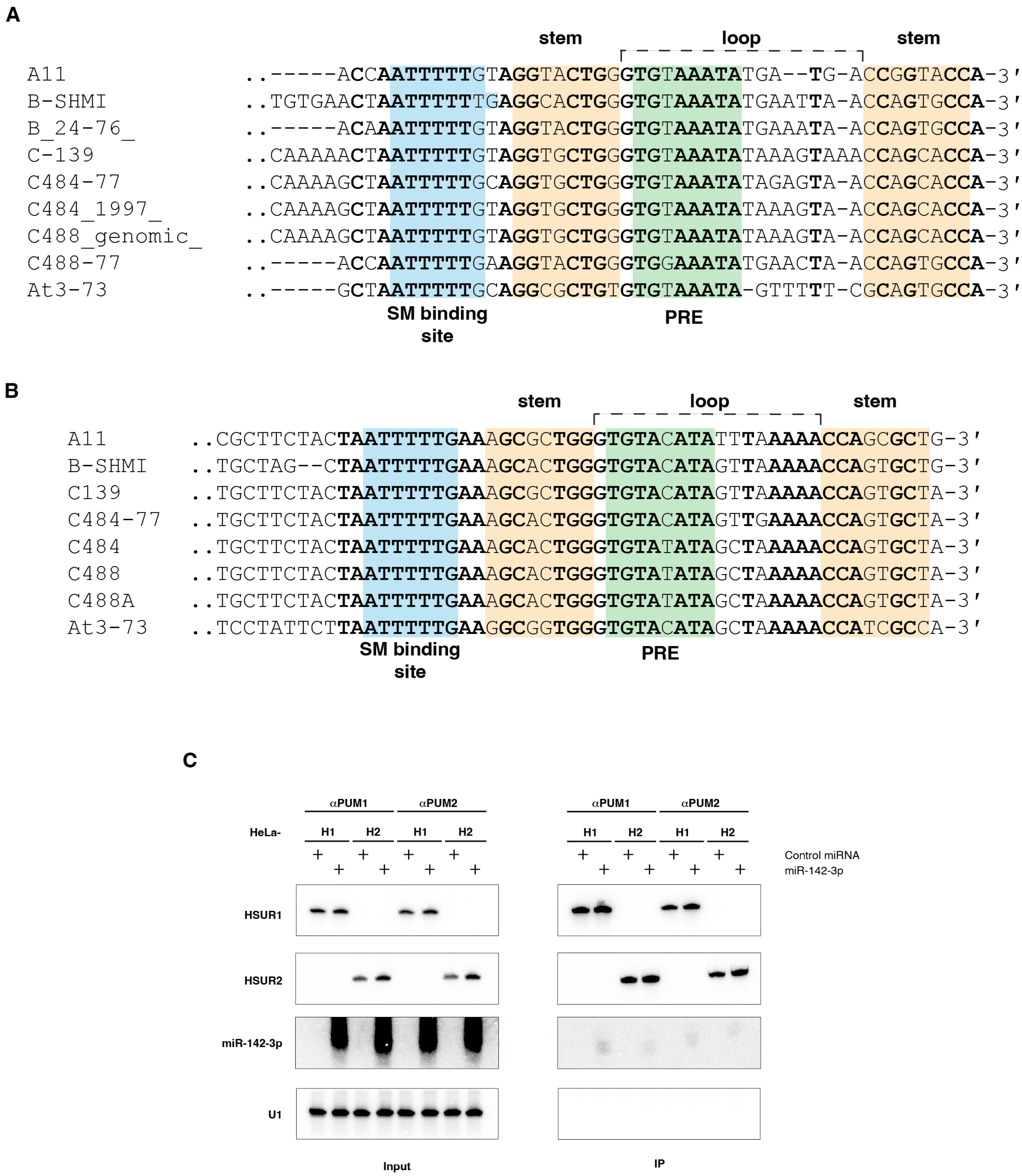

Supplementary Figure 1. HSUR1 and HSUR2 associate with Pumilio proteins.
(A) Alignment of reported genomic sequences (see main text) spanning the SM binding site to the 3′ end of HSUR1. The Sm binding site is highlighted in blue, sequences corresponding to stems are highlighted in orange, and sequences corresponding to the Pumilio Response Element (PRE) are highlighted in green. Perfectly conserved residues are shown in bold. **(B)** Same as in (A) for HSUR2. **(C)** Co-immunoprecipitation of HSUR1 and HSUR2 with antibodies

bioRxiv preprint doi: https://doi.org/10.64898/2026.10.06.757068; this version posted October 7, 2026. The copyright holder for this preprint (which was not certified by peer review) is the author/funder, who has granted bioRxiv a license to display the preprint in perpetuity. It is made available under a CC-BY 4.0 International license.

against PUM1 or PUM2 from extracts prepared from HeLa cells expressing either HSUR1 (HeLa-H1) or HSUR2 (HeLa-H2) following transfection with either a control miRNA or miR-142-3p. Northern blotting was used to detect HSUR1, HSUR2, miR-142-3p, and cellular U1 snRNA as control for immunoprecipitation and sample loading. Input, 5%; IP, 95%.

bioRxiv preprint doi: https://doi.org/10.64898/2026.10.06.757068; this version posted October 7, 2026. The copyright holder for this preprint (which was not certified by peer review) is the author/funder, who has granted bioRxiv a license to display the preprint in perpetuity. It is made available under a CC-BY 4.0 International license.

Supplementary Figure 2

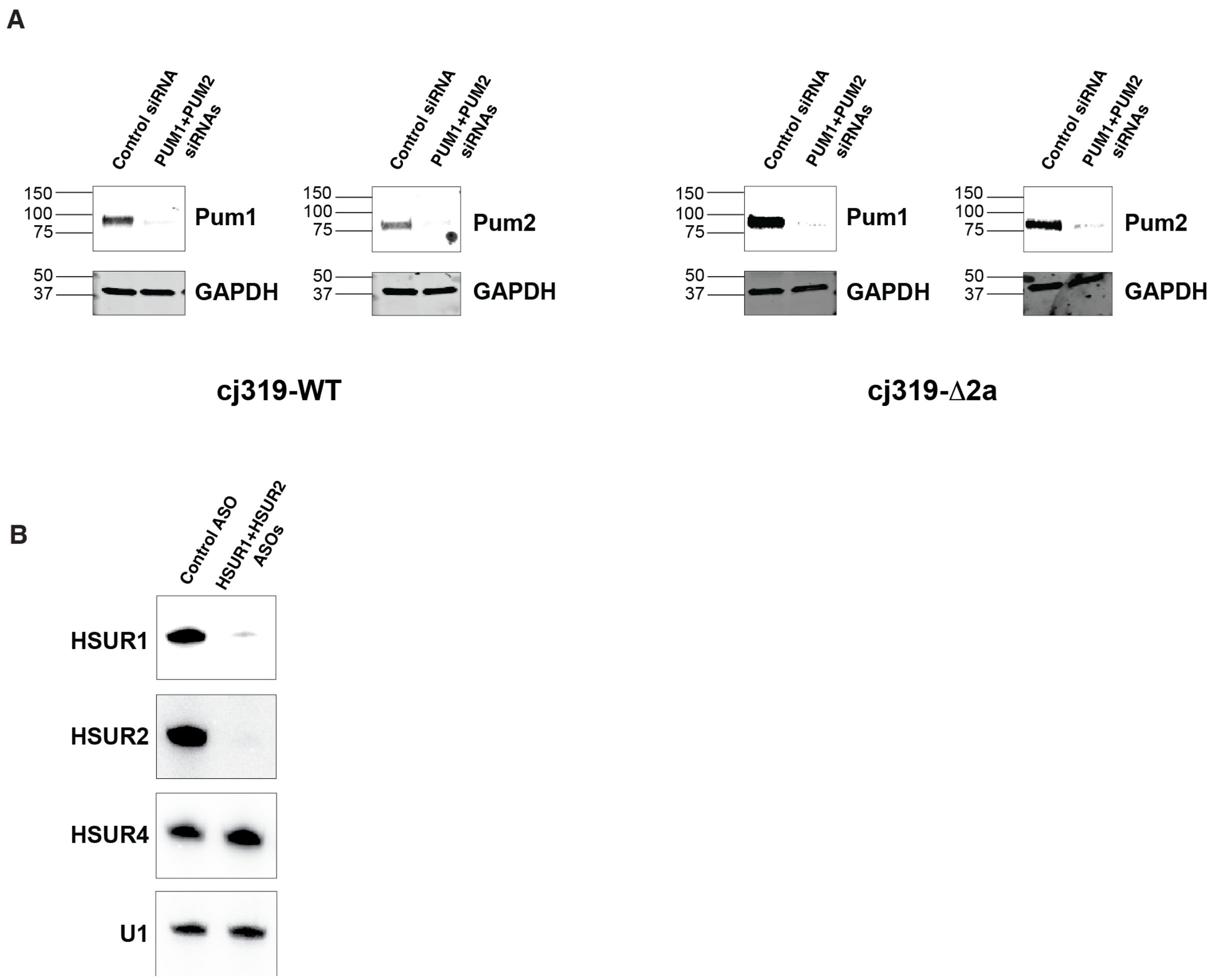

Supplementary Figure 2. Knockdown of PUM proteins and HSUR expression in HVS-infected cells.

(A) Western blot analyses of PUM1 and PUM2 in extracts from cj319-WT and cj319-Δ2a cells transiently transfected with either a control siRNA or siRNAs targeting PUM1 and PUM2. GAPDH serves as a loading control. (**B)** Northern blot analysis of HSUR1, HSUR2, HSUR4, and cellular U1 snRNAs in total RNA from cj319-WT cells transiently transfected with either a control ASO or ASOs targeting HSUR1 and HSUR2.

bioRxiv preprint doi: https://doi.org/10.64898/2026.10.06.757068; this version posted October 7, 2026. The copyright holder for this preprint (which was not certified by peer review) is the author/funder, who has granted bioRxiv a license to display the preprint in perpetuity. It is made available under a CC-BY 4.0 International license.

Supplementary Figure 3

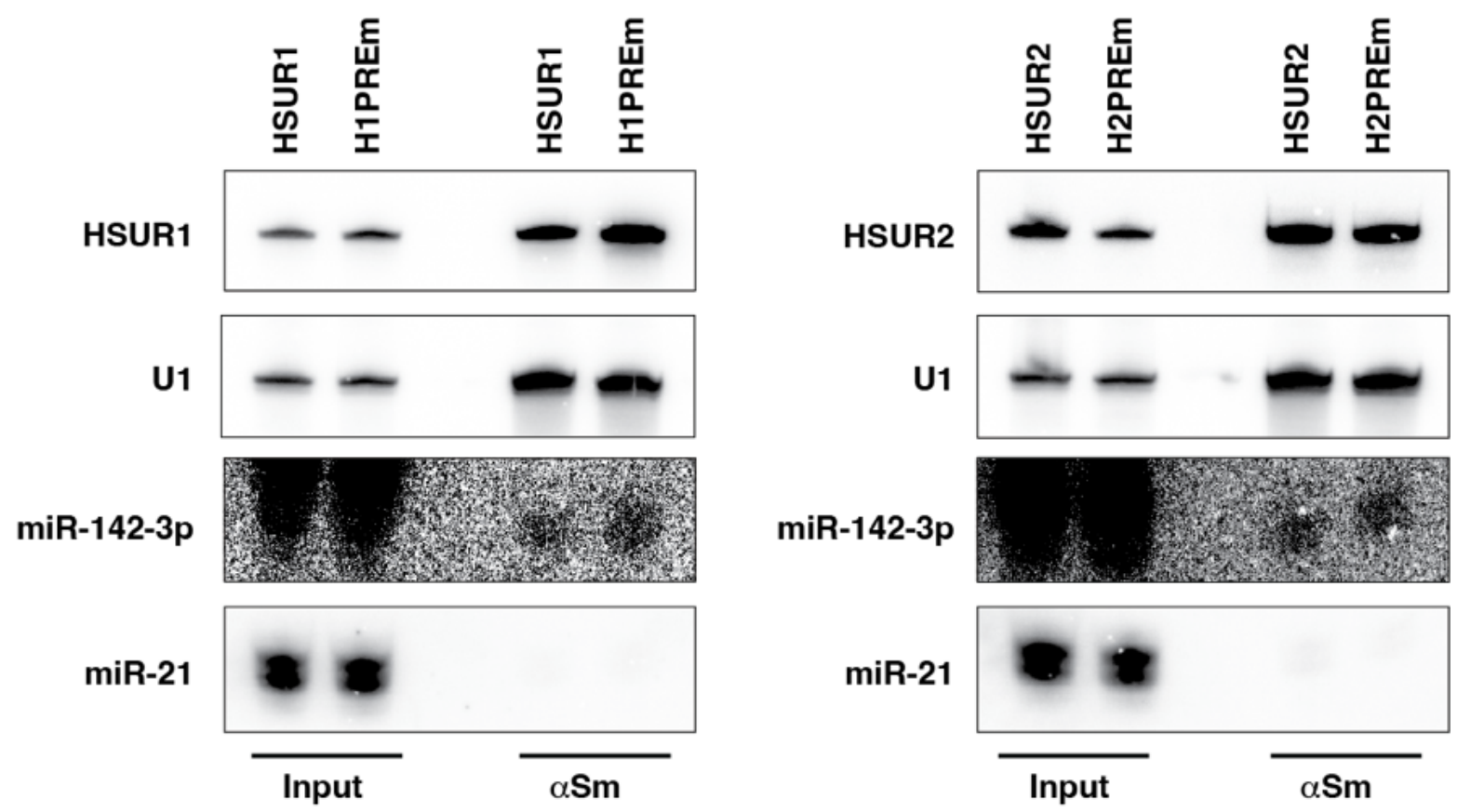

Supplementary Figure 3. PUM proteins are not required for HSUR-miR-142-3p association.

Co-immunoprecipitation of miRNAs with αSm antibodies from HeLa-H1, HeLa-H1PREm, HeLa-H2, and HeLa-H2PREm cells following transfection with miR-142-3p. Northern blot was probed for HSUR1, HSUR2, miR-142-3p and U1 snRNA as immunoprecipitation control. Input, 5%; IP, 95%.

bioRxiv preprint doi: https://doi.org/10.64898/2026.10.06.757068; this version posted October 7, 2026. The copyright holder for this preprint (which was not certified by peer review) is the author/funder, who has granted bioRxiv a license to display the preprint in perpetuity. It is made available under a CC-BY 4.0 International license.

Supplementary Figure 4

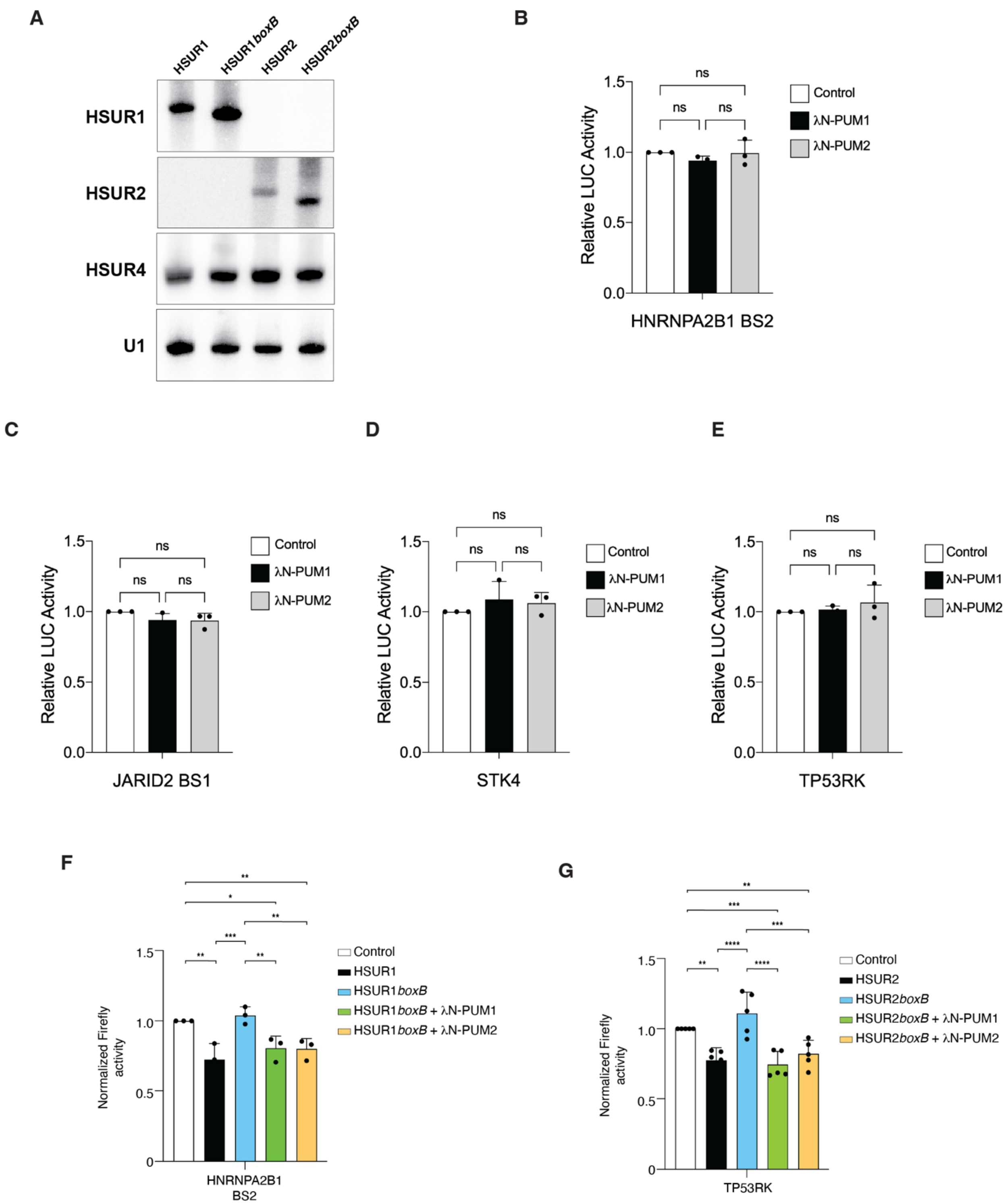

Supplementary Figure 4. Association of PUM proteins with HSURs is required for HSUR-mediated mRNA repression.

bioRxiv preprint doi: https://doi.org/10.64898/2026.10.06.757068; this version posted October 7, 2026. The copyright holder for this preprint (which was not certified by peer review) is the author/funder, who has granted bioRxiv a license to display the preprint in perpetuity. It is made available under a CC-BY 4.0 International license.

(A) Northen blot analyses of HeLa cells following transient co-transfection with a plasmid expressing HSUR4 and the plasmids used in Fig. 4 to express wild-type HSUR1 (143 nt), HSUR1*boxB* (137 nt), wild-type HSUR 2 (115 nt), or HSUR2*boxB* (107 nt). Membranes were probed for the indicated RNAs. HSUR4 served as a transfection control, whereas cellular U1 snRNA served as a loading control. **(B-E)** Luciferase assays in HuT78 cells stably expressing reporter transcripts containing 3′UTR fragments with single HSUR1-binding sites (B and C), or U937 cells stably expressing reporter transcripts containing 3′UTR fragments with single HSUR2-binding sites (D and E) following transient co-transfection of a control plasmid expressing mCherry2 alone (Control) or plasmids encoding mCherry2 and λN-PUM1 or λN-PUM2, together with a control plasmid containing the U1 promoter. Firefly luciferase levels were normalized to *Renilla* luciferase levels. **(F)** Same as in Fig. 4B with a reporter transcript containing a fragment of HNRNPA2B1 3′UTR with a single HSUR1 binding site (binding site 2, BS2). **(G)** Same as in Fig. 4C, using a reporter transcript containing a fragment of the TP53RK 3′UTR with a single HSUR2 binding site. *$P < 0.05$; **$P < 0.01$; ***$P < 0.001$; ****$P < 0.0001$.

bioRxiv preprint doi: https://doi.org/10.64898/2026.10.06.757068; this version posted October 7, 2026. The copyright holder for this preprint (which was not certified by peer review) is the author/funder, who has granted bioRxiv a license to display the preprint in perpetuity. It is made available under a CC-BY 4.0 International license.

Supplementary Figure 5

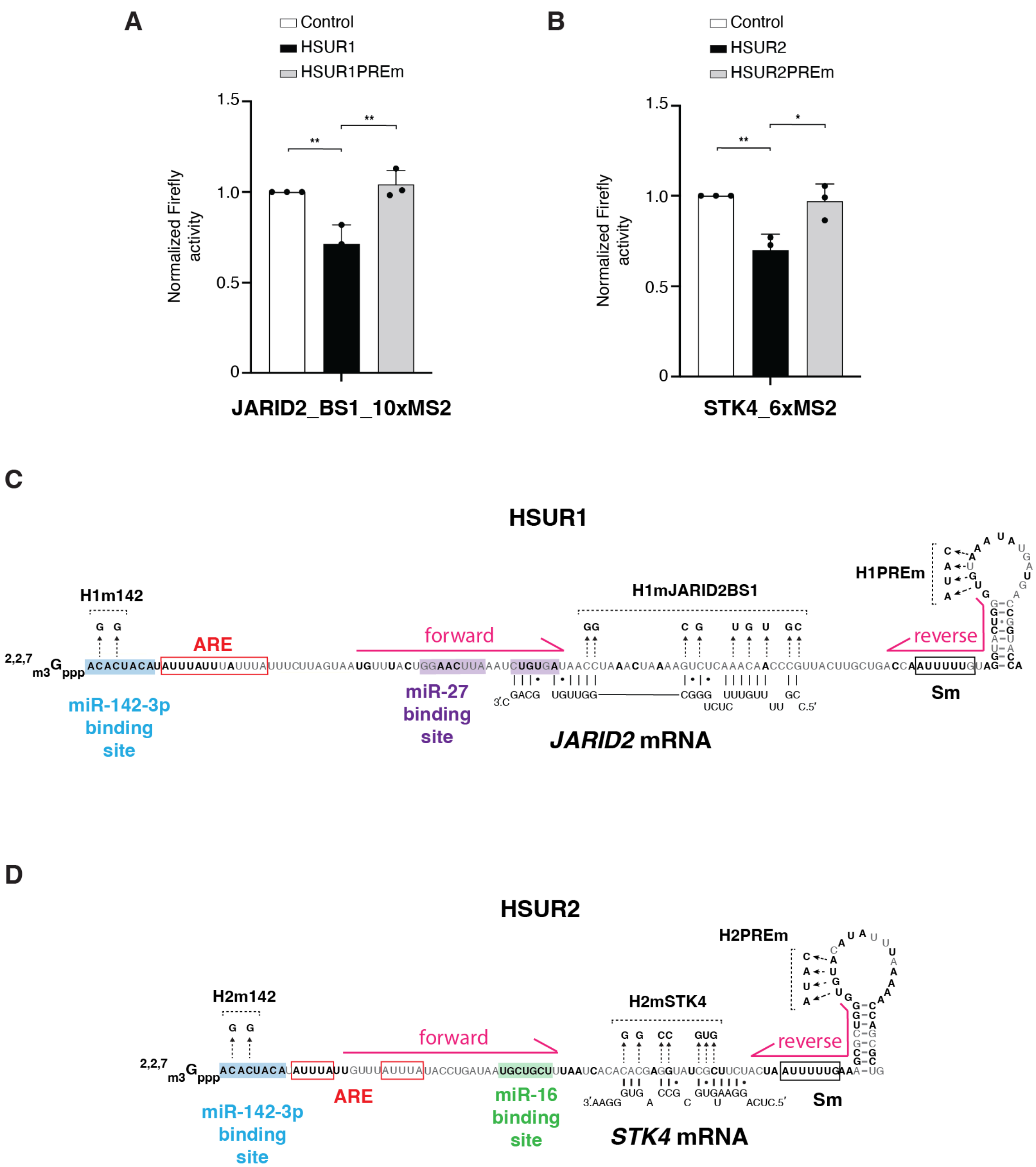

Supplementary Figure 5. PUM proteins license HSUR association with target mRNAs. **(A)** Same as in Fig. 3C, using a reporter transcript containing a fragment of JARID2 3′UTR with a single HSUR1 binding site and ten MS2 hairpin repeats. **(B)** Same as Fig. 3D, using a reporter transcript containing a fragment of the STK4 3′UTR with a single HSUR2 binding site and six MS2 hairpin repeats. **(C)** Nucleotide sequence of HSUR1 showing the nucleotides modified in

bioRxiv preprint doi: https://doi.org/10.64898/2026.10.06.757068; this version posted October 7, 2026. The copyright holder for this preprint (which was not certified by peer review) is the author/funder, who has granted bioRxiv a license to display the preprint in perpetuity. It is made available under a CC-BY 4.0 International license.

each mutant used in Fig. 5B. The position of the sequences complementary to the forward and reverse primers used to analyze HSUR1 enrichment performed in Fig. 5B are indicated by pink arrows. **(D)** Same as in (C) for HSUR2, showing the nucleotides modified in each of the mutants used in Fig. 5C and the primers used to analyze HSUR2 enrichment.
